# Treatment-related damage in elderly-onset ANCA-associated vasculitis: safety outcome analysis of two nationwide prospective cohort studies

**DOI:** 10.1186/s13075-020-02341-6

**Published:** 2020-10-12

**Authors:** Ken-Ei Sada, Keiji Ohashi, Yosuke Asano, Keigo Hayashi, Michiko Morishita, Haruki Watanabe, Yoshinori Matsumoto, Shouichi Fujimoto, Yoshinari Takasaki, Kunihiro Yamagata, Shogo Banno, Hiroaki Dobashi, Koichi Amano, Masayoshi Harigai, Yoshihiro Arimura, Hirofumi Makino, the Japan Research Committee of the Ministry of Health, Labour, and Welfare for Intractable Vasculitis (JPVAS) and the Research Committee of Intractable Renal Disease of the Ministry of Health, Labour, and Welfare of Japan, Joichi Usui, Tatsuya Atsumi, Takahiko Sugihara, Seiichi Matsuo, Hitoshi Sugiyama, Akihiro Ishizu, Takao Fujii, Yasunori Okada, Sakae Homma, Naotake Tsuboi, Shunichi Kumagai, Eri Muso, Yohko Murakawa, Shogo Banno, Hitoshi Hasegawa, Wako Yumura, Hiroaki Matsubara, Masaharu Yoshida, Kensei Katsuoka, Noriyoshi Ogawa, Atsushi Komatsuda, Satoshi Ito, Atsushi Kawakami, Izaya Nakaya, Takao Saito, Takafumi Ito, Nobuhito Hirawa, Masahiro Yamamura, Masaaki Nakano, Kosaku Nitta, Makoto Ogura, Taio Naniwa, Shoichi Ozaki, Junichi Hirahashi, Tatsuo Hosoya, Takashi Wada, Satoshi Horikoshi, Yasushi Kawaguchi, Taichi Hayashi, Tsuyoshi Watanabe, Daijo Inaguma, Kazuhiko Tsuruya, Noriyuki Homma, Tsutomu Takeuchi, Naoki Nakagawa, Shinichi Takeda, Ritsuko Katafuchi, Masayuki Iwano, Masaki Kobayashi

**Affiliations:** 1 Department of Clinical Epidemiology, Kochi Medical School, Kochi University, Kohasu, Oko-cho, Nankoku, 783-8505 Japan; 2 Department of Nephrology, Rheumatology, Endocrinology, and Metabolism, Okayama University Graduate School of Medicine, Dentistry, and Pharmaceutical Sciences, Okayama, Japan; 3 Department of Hemovascular Medicine and Artificial Organs, Faculty of Medicine, University of Miyazaki, Miyazaki, Japan; 4 Department of Internal Medicine and Rheumatology, Juntendo University School of Medicine, Tokyo, Japan; 5 Department of Nephrology, Faculty of Medicine, University of Tsukuba, Ibaraki, Japan; 6 Department of Nephrology and Rheumatology, Aichi Medical University, Nagakute, Japan; 7 Division of Hematology, Rheumatology and Respiratory Medicine, Department of Internal Medicine, Faculty of Medicine, Kagawa University, Kagawa, Japan; 8 Department of Rheumatology and Clinical Immunology, Saitama Medical Center, Saitama Medical University, Kawagoe, Japan; 9 Department of Rheumatology, Tokyo Women’s Medical University School of Medicine, Tokyo, Japan; 10 Department of Nephrology and Rheumatology, Kyorin University School of Medicine, Tokyo, Japan; 11 Kichijoji Asahi Hospital, Tokyo, Japan; 12 Okayama University, Okayama, Japan

**Keywords:** ANCA-associated vasculitis, Chronic damage, Elderly patients, Glucocorticoids

## Abstract

**Background:**

It is not elucidated that there is treatment-related damage in elderly patients with antineutrophil cytoplasmic antibody (ANCA)–associated vasculitis (AAV).

**Methods:**

Elderly (≥ 75 years of age) patients were enrolled from two nationwide prospective inception cohort studies. The primary outcome was 12-month treatment-related Vasculitis Damage Index (VDI) score. Secondary outcomes included serious infections within 6 months, total VDI score, remission, and relapse. Patient characteristics and outcomes were compared across three different initial glucocorticoid (GC) dose groups: high-dose, prednisolone (PSL) ≥ 0.8 mg/kg/day; medium-dose, 0.6 ≤ PSL < 0.8 mg/kg/day; and low-dose, PSL < 0.6 mg/kg/day.

**Results:**

Of the 179 eligible patients, the mean age was 80.0 years; 111 (62%) were female. The mean Birmingham Vasculitis Activity Score was 16.1. Myeloperoxidase-ANCA findings were positive in 168 (94%) patients, while proteinase 3-ANCA findings were positive in 11 (6%). The low-dose group was older and had higher serum creatinine levels than the other groups. There were no statistically significant intergroup differences in remission or relapse, whereas serious infection developed more frequently in the high-dose (29 patients [43%]) than the low-dose (13 patients [22%]) or medium-dose (10 patients [19%]) groups (*p* = 0.0007). Frequent VDI items at 12 months included hypertension (19%), diabetes (13%), atrophy and weakness (13%), osteoporosis (8%), and cataracts (8%). Logistic regression analysis revealed that GC dose at 12 months (odds ratio, 1.14; 95% confidence interval, 1.00–1.35) was a predictor for diabetes.

**Conclusion:**

A reduced initial GC dose with rapid reduction might be required to ensure the safe treatment of elderly AAV patients.

## Introduction

Treatment with high-dose glucocorticoid (GC) and immunosuppressants has greatly improved the prognosis of patients with antineutrophil cytoplasmic antibody (ANCA)–associated vasculitis (AAV) [1, 2], but chronic damage has become a major concern in such patients. Because disease severity was reportedly related to chronic damage [3], intensive immunosuppressive treatment is required to induce the remission of AAV. On the other hand, GC is a risk factor for chronic damage as well as infectious complications [4, 5].

AAV often occurs in elderly populations, particularly in Japan [6], and aging is a strong risk factor for death and end-stage renal disease [7–10]. Due to the high incidence of chronic conditions including diabetes mellitus, osteoporosis, cataracts, and hypertension in elderly populations with AAV [11], GC-related damage might be more serious. Because many studies of AAV have excluded elderly patients, optimization of the initial GC dose and tapering speed might be required to ensure their safe treatment.

In this study, treatment-related damage was evaluated in patients with elderly-onset AAV based on safety outcome analysis using data from two nationwide prospective inception cohort studies.

## Methods

### Database

We used datasets from two cohort studies: RemIT-JAV (observational cohort study of remission induction therapy in Japanese patients with ANCA-associated vasculitis [UMIN000001648]) and RemIT-JAV-RPGN (observational cohort study of remission induction therapy in Japanese patients with ANCA-associated vasculitis and rapidly progressive glomerulonephritis [UMIN000005136]). Consecutive patients with newly diagnosed AAV were enrolled in RemIT-JAV between April 2009 and December 2010 from 22 tertiary care institutions or in RemIT-JAV-RPGN from April 2011 to March 2014 from 53 tertiary care institutions. The criteria for enrolment for both studies included the following: (1) diagnosis of AAV by the site investigators, (2) fulfillment of criteria for primary systemic vasculitis as proposed by the European Medicines Agency algorithm [12], and (3) starting immunosuppressive treatment based on site investigator discretion [13, 14]. The exclusion criteria were as follows: (1) age younger than 20 years, (2) serologic evidence of hepatitis B or C virus infection, and (3) a history of malignancy. Baseline data recorded for each patient included demographic information, laboratory data, Birmingham Vasculitis Activity Score (BVAS) 2003 [15], and disease severity. Disease severity was subclassified as localized, early systemic, generalized, or severe according to the European Vasculitis Study Group definition of disease severity types [16]. Patients with threatened vital organ function were classified as having generalized disease, while those with organ failure were classified as having severe disease. Detailed definitions of disease severity were described previously [17]. Patients were evaluated at 3, 6, 12, 18, and 24 months and at relapse, and the following data were collected: vital status, BVAS 2003, laboratory data, treatments, and adverse events. The Vasculitis Damage Index (VDI) score was recorded at 6, 12, and 24 months [18].

### Patient selection and outcome measures

In the present study, patients with elderly-onset (≥ 75 years) AAV were enrolled. Patients for whom data about the GC dose were lacking were excluded.

The primary outcome measure was 12-month treatment-related VDI score. Treatment-related VDI was defined as in the previous report as follows: osteoporosis, diabetes, cataracts, atrophy and weakness, malignancy, gonadal failure, marrow failure, chemical cystitis, avascular necrosis, hypertension, angina/coronary artery disease, alopecia, cerebrovascular accident, myocardial infarction, and mouth ulcers [4]. Secondary outcome measures included serious infections within 6 months, 12-month total VDI score, remission, and relapse. Our definition of serious infections was based on an International Conference on Harmonization report [19]. Bacterial infections requiring intravenous antibiotic administration and opportunistic infections were considered serious infections. The diagnosis of infection was based on the attending physician’s clinical diagnosis using a comprehensive evaluation of physical findings, laboratory data, and imaging data. Remission was defined as a BVAS score of 0 on 2 consecutive occasions at least 1 month apart according to the European League Against Rheumatism (EULAR) recommendations [16]. Relapse was defined as recurrent or new-onset clinical signs and symptoms attributable to active vasculitis as we previously reported [20].

### Statistical analysis

Clinical characteristics are presented as mean ± standard deviation (SD). The patients were divided into three groups according to initial GC dose: high-dose, prednisolone (PSL) ≥ 0.8 mg/kg/day; medium-dose, 0.6 ≤ PSL < 0.8 mg/kg/day; and low-dose, PSL < 0.6 mg/kg/day. Patient characteristics and outcomes were compared across the three GC dose groups. Continuous variables were compared using the Mann–Whitney *U* test, whereas categorical variables were compared between two groups using the Fisher exact probability test. *p* values < 0.05 were considered significant. Statistical significance was determined using Bonferroni correction, < 0.05/3, to adjust for multiple testing. To explore the factors related to VDI items, multiple linear regression and logistic regression analyses were performed. All statistical analyses were performed using JMP 11.2.0 software (SAS Institute Inc., Cary, NC, USA).

## Results

### Patient characteristics

Of 477 patients registered in the two cohort studies, 181 fulfilled the inclusion criteria. Among them, 2 were excluded because of a lack of data about the GC dose. For the analysis of VDI score, 60 patients without a 12-month score were excluded. Of those 60 excluded patients, 19 died by 12 months. The mean age (SD) of the 179 enrolled patients was 80.0 (3.8) years, 111 (62%) were female, and the baseline mean (SD) BVAS was 16.1 (6.6). Seven (4%) patients had eosinophilic granulomatosis with polyangiitis, 28 (16%) had granulomatosis with polyangiitis, 113 (63%) had microscopic polyangiitis, and 31 (17%) were unclassifiable. Myeloperoxidase–antineutrophil cytoplasmic antibody (MPO-ANCA) results were positive in 168 (94%) patients, while proteinase 3–ANCA (PR3-ANCA) results were positive in 11 (6%) patients. Concomitant cyclophosphamide (CY) was used in 54 (30%) patients, and the mean (SD) GC dose was 0.73 (0.25) mg/kg/day. Prophylaxis against *Pneumocystis* pneumonia was administrated in 153 of 178 (86%) patients (there was no statistical difference compared to patients aged < 75 years: 243 of 292 [83%], *p* = 0.43).

The patient characteristics of the three groups are summarized in Table 1. The low-dose group had a significantly higher mean age than the high-dose group. There were no significant intergroup differences in sex, disease classification, or disease severity. Mean BVAS score did not differ significantly, while the high-dose group exhibited a significantly lower mean serum creatinine level than the low-dose group.

**Table 1:** Patient characteristics by initial GC dose group

	Low-dose (*n* = 59)	Medium-dose (*n* = 52)	High-dose (*n* = 68)
Male/female	27/32	18/34	23/45
Age (years), mean ± SD (median)^†^	80.9 ± 3.9 (80)	79.9 ± 3.6 (80)	79.1 ± 3.6 (78)
Disease classification, *n*			
EGPA	2	2	3
GPA	5	9	14
MPA	42	33	38
Unclassifiable	10	8	13
Disease severity, *n* (%)			
Localized	1	3	3
Early systemic	12	14	18
Systemic	34	27	35
Severe	12	8	12
BVAS, mean ± SD	15.8 ± 6.3	17.3 ± 6.0	15.4 ± 7.2
MPO-ANCA–positive, *n* (%)	55 (93)	49 (94)	64 (94)
PR3-ANCA–positive, *n* (%)	0	2 (4)	3 (4)
Serum creatinine (mg/dL), mean ± SD^†^	3.11 ± 3.56	2.36 ± 2.15	1.59 ± 1.31
Interstitial lung disease, *n* (%)	29 (49)	24 (46)	29 (43)
Treatment			
Initial daily dose of PSL (mg/kg/day), mean ± SD^#, †^	0.47 ± 0.10	0.69 ± 0.05	0.98 ± 0.18
Cyclophosphamide, *n* (%)	8 (13)	10 (19)	36 (53)

Comparisons among groups were made using the Mann–Whitney *U* test or Fisher’s exact probability test. Statistical significance was determined by using Bonferroni correction (< 0.05/3). *ANCA* antineutrophil cytoplasmic antibody, *BVAS* Birmingham Vasculitis Activity Score, *EGPA* eosinophilic granulomatosis with polyangiitis, *GPA* granulomatosis with polyangiitis, *MPA* microscopic polyangiitis, *MPO* myeloperoxidase, *PR3* proteinase 3, *PSL* prednisolone, *SD* standard deviation

^#^Medium-dose vs. high-dose group

^†^Low-dose vs. high-dose group

Mean (SD) daily PSL doses at initial treatment in the low-, medium-, and high-dose groups were 0.47 (0.10), 0.69 (0.05), and 0.98 (0.18) mg/kg, respectively. The proportion of concomitant CY use was significantly different among groups: low-dose, 8 (13%); medium-dose, 10 (19%); and high-dose, 36 (53%); *p* < 0.0001.

### Treatment effectiveness and safety

**Fig. 1:**
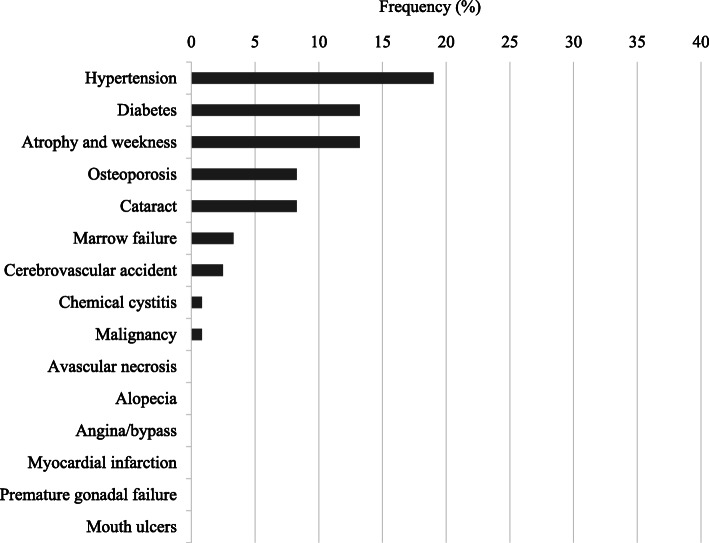
Frequency of treatment-related VDI items at 12 months. VDI, Vasculitis Damage Index

By 12 months, 151 (84%) patients achieved remission; of those, 17 (11%) experienced a relapse. Mean (SD) total VDI score at 12 months was 2.4 (1.8), while mean (SD) treatment-related VDI score was 0.7 (1.0). The proportions of patients with each VDI item at 12 months are shown in Fig. 1. Frequent VDI items (> 5% of enrolled patients) were as follows: hypertension (19%), diabetes (13%), atrophy and weakness (13%), osteoporosis (8%), and cataracts (8%). Serious infections within 6 months developed in 52 (29%) patients.

There were no statistically significant intergroup differences in remission or relapse by 12 months: remission—50 (85%) of 59 patients in low-dose, 41 (79%) of 52 in medium-dose, and 60 (88%) of 68 in high-dose, respectively, *p* = 0.38; relapse—5 (10%) of 50 in low-dose, 5 (12%) of 41 in medium-dose, and 7 (12%) of 60 in high-dose, respectively, *p* = 0.93. Even limited in patients with severe form, remission and relapse were not different statistically (remission: 66% in low-dose, 63% in medium-dose, and 67% in high-dose, respectively, *p* = 0.98; relapse: 0%, 20%, and 13%, respectively, *p* = 0.46). Serious infections developed more frequently in the high-dose (*n* = 29 [43%]) than in the low-dose (*n* = 13 [22%]) or medium-dose (*n* = 10 [19%]) groups (*p* = 0.0007). Same trends were found in each dataset (RemIT-JAV group: 45% in high-dose, 13% in medium-dose, and 15% in low-dose, respectively, *p* = 0.04; RemIT-JAV-RPGN group: 41% in high-dose, 22% in medium-dose, and 24% in low-dose, respectively, *p* = 0.10).

### Vasculitis Damage Index

Mean (SD) VDI scores of all patients in the RemIT-JAV and RemIT-JAV-RPGN groups were 1.7 (1.7) and 2.1 (1.6) at 6 months, and 2.0 (1.7) and 2.5 (1.8) at 12 months, respectively.

Of 121 patients with VDI scores at 12 months, total and treatment-related VDI scores at 12 months showed no statistically significant intergroup differences (total VDI score: low-dose, 2.65 ± 1.99; medium-dose, 2.40 ± 1.67; and high-dose, 2.25 ± 1.70, *p* = 0.57; treatment-related VDI score: low-dose, 0.91 ± 1.18; medium-dose, 0.45 ± 0.71; and high-dose, 0.77 ± 1.04, *p* = 0.14).

For the determination of variables associated with total and treatment-related VDI scores at 12 months, a multiple linear regression analysis was performed of the following candidate variables: sex, age, initial GC dose, initial CY usage, and initial serum creatinine level. No statistically significant variables were identified (Supplementary Table 1).

Next, we explored variables related to 5 frequent treatment-related VDI items at 12 months using logistic regression analysis including the same candidate variables. Hypertension, atrophy and weakness, osteoporosis, and cataracts were eliminated (Supplementary Table 2), while CY usage was identified as an independent predictor for a less frequent development of diabetes (Table 2, model 1).

**Table 2:** Risk factors for the development of diabetes at 12 months

	Odds ratio (95% CI)	*p* value
Model 1		
Age, years	1.06 (0.93–1.24)	0.36
Female sex	0.73 (0.24–2.01)	0.55
Serum creatinine, mg/dL	0.83 (0.66–0.99)	0.040
Initial PSL dose, mg/kg/day	3.32 (0.29–41.66)	0.34
Concomitant cyclophosphamide use	0.29 (0.09–0.87)	0.027
Model 2		
Age, years	1.08 (0.94–1.26)	0.28
Female sex	0.76 (0.25–2.09)	0.60
Serum creatinine, mg/dL	0.86 (0.70–1.02)	0.09
Initial PSL dose, mg/kg/day	1.26 (0.13–14.65)	0.85
PSL dose at 12 months, mg/day	1.14 (1.00–1.35)	0.045

*CI* confidential interval, *PSL* prednisolone

Because mean (SD) GC dose at 12 months was lower in patients with concomitant CY than in those without it (6.8 [3.5] vs. 9.5 [6.9] mg/day, *p* = 0.016), we performed a multiple linear regression analysis including GC dose at 12 months instead of concomitant CY usage; GC dose at 12 months was also identified as an independent predictor for diabetes (Table 2, model 2).

## Discussion

This is the first report to evaluate damage in elderly patients with AAV. The low-dose initial GC group was older and had more severe renal impairment but less frequent concomitant CY use. The high-dose group more frequently developed serious infections. Total and treatment-related VDI scores did not differ significantly among the initial GC dose groups. GC dose at 12 months was an independent predictor for diabetes.

Our findings suggest that initial GC dose perhaps is reduced during treatment in elderly patients with AAV. Serious infections developed more frequently in the high-dose group, but remission, relapse, and VDI score were comparable to those of the other groups. Although CY dose was recommended to be reduced according to age and renal function with concern about adverse drug reactions in the EULAR recommendation for the treatment of AAV [21], the initial dose adjustment of GC was not stated, and PSL 1 mg/kg/day has traditionally been initiated since the 1970s [22]. The PEXIVAS trial reported that a reduced-dose GC regimen (initial dose, ~ 0.5 mg/kg/day) was non-inferior to the standard regimen (initial dose, ~ 1 mg/kg/day) with respect to death or end-stage renal failure in patients with severe AAV [23]. Initial GC dose and age were reportedly related to serious infection in the immunosuppressive treatment of rheumatic diseases [24–26]. Our previous study also showed that PSL > 0.8 mg/kg/day was a risk factor for severe infection in patients with AAV [5]. Therefore, the reduction of the initial GC dose could lead to improved safety outcomes in elderly patients.

Diabetes, atrophy and weakness, osteoporosis, and cataracts were emphasized as chronic damage in elderly AAV patients. Although the order of the frequency of treatment-related VDI items was comparable to those of the previous report for VDI for AAV patients of all ages, diabetes (13% in present study vs. 8% in the previous study), atrophy and weakness (13% vs. 6%), osteoporosis (8% vs. 4%), and cataracts (8% vs. 3%) were more frequent in the present study [27]. These items are also well-known concerns in general elderly patients, and our result indicates that AAV treatment accelerates this damage in elderly AAV patients.

A delay in the GC reduction without concomitant CY usage and renal impairment might lead to the development of diabetes. Concomitant CY usage was determined as a protective predictor for diabetes by analysis of initial treatment with patient characteristics. Our recent study also demonstrated a GC sparing effect of concomitant CY in AAV patients, as the GC dose at 12 months was lower in patients with concomitant CY than those without CY [28]. Even on multivariate analysis with the model including GC dose at 12 months instead of concomitant CY usage, GC dose at 12 months remained a statistical predictor for diabetes. Because the achievement of PSL 5–7.5 mg/day by 5 months is recommended in the EULAR guidelines, an earlier reduction of GC dose might be attempted in elderly AAV patients with renal impairment [21].

There are some limitations to this study. First, the majority of our patients were MPO-ANCA–positive. There are several differences in characteristics between patients with MPO-ANCA and those with PR3-ANCA [29, 30], and our results might not be applicable to the treatment of elderly patients with PR3-ANCA. Second, the treatment strategy was decided by each attending physician, so frail patients might have been treated conservatively, leading to underestimation of the outcomes. Nevertheless, patients with higher GC doses at 12 months had diabetes more frequently, supporting the relevance of a sufficient and early GC dose reduction in elderly patients. Third, we could not validate our exploratory results in another dataset because of insufficient sample size, so further validation study is required.

## Conclusion

Rapidly reducing the initial GC dose to PSL < 0.8 mg/kg/day perhaps is required to ensure the safe treatment of elderly patients with AAV.

## Supplementary information

**Additional file 1: Table S1.** Risk factors for total and treatment-related VDI. **Table S2.** Risk factors for hypertension, atrophy and weakness, cataract, and osteoporosis.

## Data Availability

The dataset analyzed in this paper is available from the corresponding author on reasonable request.

## Abbreviations

AAV: Antineutrophil cytoplasmic antibody–associated vasculitis
ANCA: Antineutrophil cytoplasmic antibody
BVAS: Birmingham Vasculitis Activity Score
CY: Cyclophosphamide
EULAR: European League Against Rheumatism
GC: Glucocorticoid
MPO-ANCA: Myeloperoxidase–antineutrophil cytoplasmic antibody
PR3-ANCA: Proteinase 3–antineutrophil cytoplasmic antibody
PSL: Prednisolone
RemIT-JAV: Observational cohort study of remission induction therapy in Japanese patients with antineutrophil cytoplasmic antibody-associated vasculitis
RemIT-JAV-RPGN: Observational cohort study of remission induction therapy in Japanese patients with antineutrophil cytoplasmic antibody-associated vasculitis and rapidly progressive glomerulonephritis
SD: Standard deviation
VDI: Vasculitis Damage Index
