# Supplementary material for: Treatment-related damage in elderly-onset ANCA-associated vasculitis: safety outcome analysis of two nationwide prospective cohort studies
Source: Arthritis Res Ther. 2020 Oct 12;22:236. doi: 10.1186/s13075-020-02341-6 (PMC7552473; doi:10.1186/s13075-020-02341-6)
Supplement: Supplementary file 1 — Additional file 1: Table S1. Risk factors for total and treatment-related VDI. Table S2. Risk factors for hypertension, atrophy and weakness, cataract, and osteoporosis. [file 13075_2020_2341_MOESM1_ESM.docx]

**Supplementary Table 1. Risk factors for total and treatment-related VDI**

|  | Total VDI | | Treatment-related VDI | |
| --- | --- | --- | --- | --- |
|  | β-coefficient (95% CI) | *P* value | β-coefficient (95% CI) | *P* value |
| Age, years | -0.01 (-3.41–11.05) | 0.30 | 0.00 (-0.05–0.05) | 0.97 |
| Female sex | 0.03 (-0.32–0.37) | 0.88 | -0.03 (-0.22–0.17) | 0.79 |
| Serum creatinine, mg/dL | 0.10 (-0.04–0.23) | 0.16 | 0.04 (-0.04–0.12) | 0.36 |
| Initial PSL dose, mg/kg/day | -0.99 (-2.54–0.55) | 0.21 | -0.38 (-1.26–0.49) | 0.39 |
| Concomitant cyclophosphamide use | -0.02 (-0.40–0.35) | 0.91 | -0.02 (-0.23–0.19) | 0.86 |

CI, confidential interval; PSL, prednisolone; VDI, Vasculitis Damage Index

**Supplementary Table 2. Risk factors for hypertension, atrophy and weakness, cataract, and osteoporosis**

|  | Hypertension  Odds ratio (95% CI) | Atrophy and weakness  Odds ratio (95% CI) | Cataract  Odds ratio (95% CI) | Osteoporosis  Odds ratio (95% CI) |
| --- | --- | --- | --- | --- |
| Age, years | 0.92 (0.82–1.04) | 1.02 (0.90–1.18) | 0.94 (0.80–1.14) | 1.11 (0.93–1.35) |
| Female sex | 0.97 (0.38–2.58) | 0.65 (0.24–1.84) | 0.89 (0.24–3.70) | 1.09 (0.32–4.36) |
| Serum creatinine, mg/dL | 0.95 (0.80–1.14) | 0.99 (0.82–1.27) | 0.98 (0.79–1.34) | 1.17 (0.87–1.83) |
| Initial PSL dose, mg/kg/day | 6.14 (0.69–65.22) | 0.51 (0.05–5.92) | 5.38 (0.23–151.93) | 0.36 (0.02–6.83) |
| Concomitant cyclophosphamide use | 0.65 (0.19–1.95) | 0.80 (0.24–2.44) | 0.27 (0.01–1.68) | 0.24 (0.03–1.06) |

CI, confidential interval; PSL, prednisolone; VDI, Vasculitis Damage Index
